# N-linked glycosylation at site 158 of the HA protein of H5N6 highly pathogenic avian influenza virus is important for viral biological properties and host immune responses

**DOI:** 10.1186/s13567-020-00879-6

**Published:** 2021-01-13

**Authors:** Ruyi Gao, Min Gu, Liwei Shi, Kaituo Liu, Xiuli Li, Xiaoquan Wang, Jiao Hu, Xiaowen Liu, Shunlin Hu, Sujuan Chen, Daxin Peng, Xinan Jiao, Xiufan Liu

**Affiliations:** 1 College of Veterinary Medicine, Yangzhou University, No.48 East Wenhui Road, Yangzhou, 225009 Jiangsu China; 2 Jiangsu Key Laboratory of Zoonosis, Yangzhou University, Yangzhou, 225009 Jiangsu China; 3 Jiangsu Co-Innovation Center for Prevention and Control of Important Animal Infectious Diseases and Zoonosis, Yangzhou, 225009 Jiangsu China

**Keywords:** H5N6 avian influenza virus, glycosylation, pathogenicity, host immune response

## Abstract

Since 2014, clade 2.3.4.4 has become the dominant epidemic branch of the Asian lineage H5 subtype highly pathogenic avian influenza virus (HPAIV) in southern and eastern China, while the H5N6 subtype is the most prevalent. We have shown earlier that lack of glycosylation at position 158 of the hemagglutinin (HA) glycoprotein due to the T160A mutation is a key determinant of the dual receptor binding property of clade 2.3.4.4 H5NX subtypes. Our present study aims to explore other effects of this site among H5N6 viruses. Here we report that N-linked glycosylation at site 158 facilitated the assembly of virus-like particles and enhanced virus replication in A549, MDCK, and chicken embryonic fibroblast (CEF) cells. Consistently, the HA-glycosylated H5N6 virus induced higher levels of inflammatory factors and resulted in stronger pathogenicity in mice than the virus without glycosylation at site 158. However, H5N6 viruses without glycosylation at site 158 were more resistant to heat and bound host cells better than the HA-glycosylated viruses. H5N6 virus without glycosylation at this site triggered the host immune response mechanism to antagonize the viral infection, making viral pathogenicity milder and favoring virus spread. These findings highlight the importance of glycosylation at site 158 of HA for the pathogenicity of the H5N6 viruses.

## Introduction

The Asian lineage of H5N1 (H5NX) subtype highly pathogenic avian influenza virus (HPAIV) was first isolated in 1996 from diseased geese in southern China [1]. It has spread to more than 70 countries in Asia, Africa, Europe, and North America and causes great losses to the world poultry industry [1, 2]. In 1997, the first human case of H5N1 infection occurred in Hong Kong [3]. According to the WHO data, as of June 2020, a total of 861 confirmed human cases of H5N1 infection with 455 deaths have been reported in 17 countries and regions around the world. As such it has posed a major threat to public health [4]. H5 HPAIV is currently endemic in China, with multiple genotypes and subtypes being prevalent. The clade 2.3.4.4 is a new sub-branch of H5 HPAIV that appeared in 2008, and contains various subtypes such as H5N2, H5N3, H5N5, H5N6 and H5N8 [5]. Recent epidemiological surveillance data show that clade 2.3.4.4 has become the dominant epidemic branch in southern and eastern China, while H5N6 isolates have replaced the H5N1 strains as the dominant epidemic subtype [6].

The genetic composition of H5N6 HPAIV is extremely complex. It is comprised of as many as 34 genotypes, including a genotype whose internal gene cassette is entirely derived from the H9N2 avian influenza virus (AIV) [6, 7]. The H5N6 virus has a broad-spectrum host range. In addition to avian species including poultry, waterfowl, and wild birds, some mammals such as pigs and cats can also be infected [8, 9]. Furthermore, except for H5N1, the H5N6 subtype of HPAIV is currently the only H5 subtype that can infect humans [10]. As of June 2020, a total of 24 cases of human infection with H5N6 HPAIV have been reported, including eight deaths [4]. Therefore, H5N6 HPAIV poses a realistic threat to public health.

Compared to the surface Hemagglutinin (HA) glycoprotein of the precursor H5N1 HPAIV from clade 2.3.4, the vast majority of H5N6 HPAIV and human H5N6 viruses bear a T160A (H3 numbering) mutation in the HA protein, resulting in deletion of the *N*-linked glycosylation site at position 158 [11, 12]. Glycosylation of HA is associated with the receptor binding properties, transmission, pathogenicity and antigenicity of influenza viruses [13–16]. Gao et al. first found that the lack of glycosylation at site 158 enhances the binding of the HPAIV H5N1 subtype to human receptors and transmission in guinea pigs [13]. Furthermore, lack of glycosylation site 158 in combination with other nucleotide variations in the H5N1 HA protein promotes aerosol transmission between ferrets [14]. Removal of the glycosylation site at 158 in H5N1 HPAIV reduces virus replication in cells but increases pathogenicity in mice [15]. Moreover, Wang et al. showed that 158 N glycosylation of the human H5N1 virus affects virus antigenicity and replication by masking the antigenic epitopes in the HA globular head and inducing the α2,3SAL binding preference, resulting in a lower antibody response in ferrets [17]. In addition to these H5 viruses, T160A that leads to the loss of glycosylation in the *receptor-binding region* of HA has been found in human H7N9 viruses [18]. Thus, more glycosylation functions of the HA protein remain to be discovered.

Development of the disease depends on both pathogen and host defense elements. The equilibrium between the innate immune response and the pathogenicity of H5N1 influenza virus contributes to disease severity [19–21]. Hypercytokinemia often occurs in cases of human infections with H5N6 HPAIV. Patients having survived from H5N6 HPAIV infections tend to develop lower levels of circulating pro- and anti-inflammatory cytokines/chemokines [22]. Understanding the pathogenesis of these viruses will help design novel intervention strategies to prevent and treat fatal infections caused by H5N6 viruses.

Our recent study showed that lack of glycosylation at position 158 of the HA protein due to the T160A mutation affects the dual receptor binding of clade 2.3.4.4 H5NX subtype viruses. However, the recombinant viruses lacking glycosylation at site 158 exhibit higher pathogenicity than those with glycosylation at site 158, though both share the same PR8H1N1 (human influenza A virus) internal gene cassette [23]. Our present study is aimed at understanding the effect of glycosylation in the HA protein on the pathogenicity of H5N6 virus and the host immune response.

## Materials and methods

### Viruses and cells

Two H5N6 avian influenza viruses, A/goose/Guangdong/Y6/2015(Y6) and A/duck/Guangdong/HX/2015(HX) were plaque-purified in Madin-Darby canine kidney (MDCK) cells for three consecutive generations before passaging in 10 day-old specific-pathogen-free (SPF) embryonic chicken eggs to acquire virus stocks as previously described [24]. MDCK cells, human embryonic (293T) cells, chicken embryo fibroblast (CEF) cells, and human type II respiratory epithelial (A549) cells were maintained in Dulbecco modified Eagle medium (DMEM) containing 10% fetal bovine serum (FBS, Gibco) at 37 °C with 5% CO_2_. All experiments with HPAI H5N6 viruses were conducted in an animal biosafety level 3 laboratory.

### Mutagenesis, virus rescue and identification

Single mutations of T to A or A to T at site 160 in the HA genes of Y6 and HX strains were generated using the Fast Mutagenesis System (TransGEN) according to the manufacturer’s instructions. The modified HA genes were cloned into the pHW2000 vector and confirmed by sequencing. Then, a mixture of 293T and MDCK cells was transfected with eight rescue plasmids with or without mutant HA plasmids using the PolyFect transfection reagent (QIAGEN) [25]. The rescued viruses were detected by a hemagglutination assay and were fully sequenced to ensure the absence of non-desired mutations. The rescued viruses were passaged three times in SPF embryonic chicken eggs to ensure no reverse mutation. To quantify virus titer, 50% tissue culture infectious dose (TCID_50_) was measured in MDCK cells and CEF cells and 50% egg infectious doses (EID_50_) was determined in 10 day-old SPF eggs.

### Antigenicity analysis

Four H5N6 viruses were tested for antigenic reactivity with a hemagglutination inhibition (HI) assay using vaccine strain Re-8 antisera, which was available for clade 2.3.4.4 virus. HI assays were performed with 1% chicken erythrocytes as described previously [26].

### Pathogenicity in mice

To determine the 50% mouse lethal dose (MLD_50_) of viruses, four groups of five 6 week-old female BALB/c mice (from the Experimental Animal Center of Yangzhou University) were inoculated intranasally with tenfold serial dilutions containing 10^3^ to 10^7^ EID_50_ of virus in a 50 µL volume. The MLD_50_ was calculated using the method of Reed and Muench [27]. Two groups of six mice were inoculated intranasally with 10^6^ EID_50_ of RY6 or RY6-160T strains in a 50 µL volume. Three mice from each group were euthanized on day 3 and three on day 5 post-infection. Organs, including the lung, kidney, spleen, heart, turbinate and brain, were collected for virus titration as described previously [15].

To determine the viral load and cytokine production in the turbinate, trachea and lung following infection with the indicated viruses, two groups of mice were infected intranasally with 10^6^ EID_50_ of RY6 or RY6-160T in a 50 µL volume. Three mice from each group were euthanized at 3, 6, 9, 12, 20, 24 and 72 h post-infection (hpi), respectively. The Taqman probe method was performed to measure virus titer and the relative transcript levels for selected cytokines were assayed by quantitative, real-time PCR as described previously [15, 28].

### Viral growth

Monolayers of CEF, A549 and MDCK cells were infected with each virus at multiplicities of infection (MOI) of 0.01 and 5 in serum-free DMEM, and supernatants were collected at 12, 24, 48, 72 and 96 hpi. Infectious virus titers were measured using the TCID_50_ assay in MDCK cells and calculated according to the Reed-Muench method [27].

### Receptor binding assay

To evaluate the receptor-binding properties of the reassortant H5 viruses, we initially performed solid-phase binding assays with the synthetic sialyl glycopolymers Neu5Aca2-3Galb1-4GlcNAcb(3ʹSLN)-PAA-biotin and Neu5Aca2-3Galb1-4GlcNAcb(6ʹSLN)-PAA-biotin (GlycoTech) as previously described [29]. Two different glycan analogs were serially diluted in PBS and added to the 96-well streptavidin-coated microtiter plates (Pierce). The plates were blocked with PBS containing 2% skim milk powder, and 64 HA units of virus were added per well. Then, chicken antiserum against the virus was added to each well as the first antibody. The reaction was detected by sequential addition of HRP-conjugated rabbit anti-chicken IgG antibody and tetramethyl benzidine substrate solution, and then stopped with 1 M H_2_SO_4_ to read the absorbance at 450 nm. Each sample was measured in triplicate.

### Virus adhesion assay

The ability of viruses to bind to host cell membranes was estimated by flow cytometry. Briefly, the virus was allowed to bind to cells at an MOI of 5 at 4 °C for 1 h. Unbound viruses were removed by three washes with PBS. Virus-bound cells were then incubated with an anti-HA rabbit polyclonal antibody (Abcam) and donkey anti-mouse Alexa Flour 488 secondary antibody (Abcam). The cell suspensions were then subjected to flow cytometry on a FACS LSRFortessa flow cytometer (BD Biosciences). Debris, clumps and dead cells were excluded using FSC-SSC. The positive cells for FITC fluorescence from 20 000 cells were documented at 488 nm.

### Thermal stability

With reference to a previous study [30], the recombinant viruses were incubated in a 56 ℃ water bath for different times (0, 5, 10, 15, 30, 60, 90, 120, 150, and 180 min). Infectivity and hemagglutination activity of heat-treated viruses were then determined with a standard hemagglutination assay with 1% chicken red blood cells and TCID_50_ titration in MDCK cells.

### Hemagglutination and Hemagglutination-Elution Assays

Hemagglutination assays were used to determine the balance between HA binding and NA activity. The viruses were transferred to 96-well U-shape plates and twofold serially diluted in 50 μL PBS. Then 50 μL 0.8% guinea pig erythrocytes were added to each well and incubated at 4 °C for 75 min, followed by 2 h at 37 °C. HA titers were collected at different temperatures.

### Virus-like particle (VLP) formation

We used a plasmid-based VLP system to construct two influenza VLP with a mutation in HA protein as described previously [31]. Briefly, 293T cells were transfected with pCAGGS plasmids expressing the viral proteins HA, NA, and M1 using Lipofectamine 2000 reagent (Invitrogen) according to the manufacturer’s instructions. At 48 h posttransfection, the released VLP in the cell supernatant were harvested by sucrose density gradient ultracentrifugation. The VLP content was assessed by measuring hemagglutination titers and the protein level was quantified by western blot analysis.

### RNA-seq analysis

A549 cells were infected with RY6 or RY6-160T at an MOI of 5. After infection for 6 h, cells were collected to extract total RNA using TRIZOL Reagent (Life technologies) according to the manufacturer’s instructions. Library construction and sequencing were performed by Shanghai Biotechnology Corporation. Changes in gene expression were screened by comparing gene transcription levels in experimental samples with those in uninfected control samples. Differentially expressed genes (DEG) were determined based on an adjusted *p*-value (*q*-value) < 0.05 and a fold-change > 2. Gene ontology (GO) term and KEGG pathway enrichment analysis were performed to investigate the biological significance of the DEG.

### Verification of RNA-Seq data by qRT-PCR

Based on the RNA-seq analysis, the mRNA levels of target genes were determined by quantitative real-time PCR (qRT-PCR). qRT-PCR was performed using ChamQ SYBR qPCR Master Mix (Vazyme) and the LightCycle 480 System (Roche). The threshold cycle value (Ct) of each sample was measured and normalized to the internal glyceraldehyde 3-phosphate dehydrogenase (*Gapdh*) control. Relative mRNA levels were calculated using the 2–ΔΔCT method.

### Statistical analysis

All statistical analyses were based on at least three independent experiments using the Student *t* test and were performed using GraphPad Prism 7 software. Differences with a P-value smaller than 0.05 were considered statistically significant.

## Results

### Glycosylation at site 158 does not affect the antigenicity of the H5N6 virus

Two H5N6 variants used in this study have distinct glycosylation sites at 158 of the HA proteins. The HX virus with a threonine (T) residue at 160 tends to form an NDT glycosylation motif in its HA protein, whereas the Y6 virus contains a non-glycosylated NDA motif. We conducted a PCR-based site-directed mutagenesis to generate several viruses with a single amino acid change at 160, including T160A in RHX (RHX-160A) and A160T in RY6 (RY6-160T). As shown in Table 1, these four viruses remained highly pathogenic. To investigate if glycosylation at 158 affected its antigenicity, we examined the reactivity of the recombinant viruses with antisera obtained from chickens immunized with the vaccine strain Re-8. There was no significant difference in HI titers among these four viruses (Table 1), suggesting that glycosylation at 158 has no effect on the antigenicity of viruses.

**Table 1:** Characteristics and antigenicity analysis of the four reassortant H5N6 HPAIVs

Viruses	HA titer (log2)	Characteristics				Antiserum HI(log2)
		MDT^a^(h)	lgEID_50_/0.1 mL	lgTCID_50_/0.1 mL (MDCK)	lgTCID_50_/0.1 mL (CEF)	Re-8^b^
RY6	7	36–48	8.83	6.5	7.625	10
RY6-160 T	8	36–48	8.75	7.5	7.83	9
RHX	8	36–48	8.17	6.83	7.5	8
RHX-160A	6	36–48	7.5	6.625	6.83	8

^a^Mean death time was calculated based on the data at the highest dilution in which all of the embryos died.

^b^Re-8 was the vaccine strain against clade 2.3.4.4 viruses in China.

### Glycosylation at site 158 enhances pathogenicity of the H5N6 virus in mice

**Figure 1:**
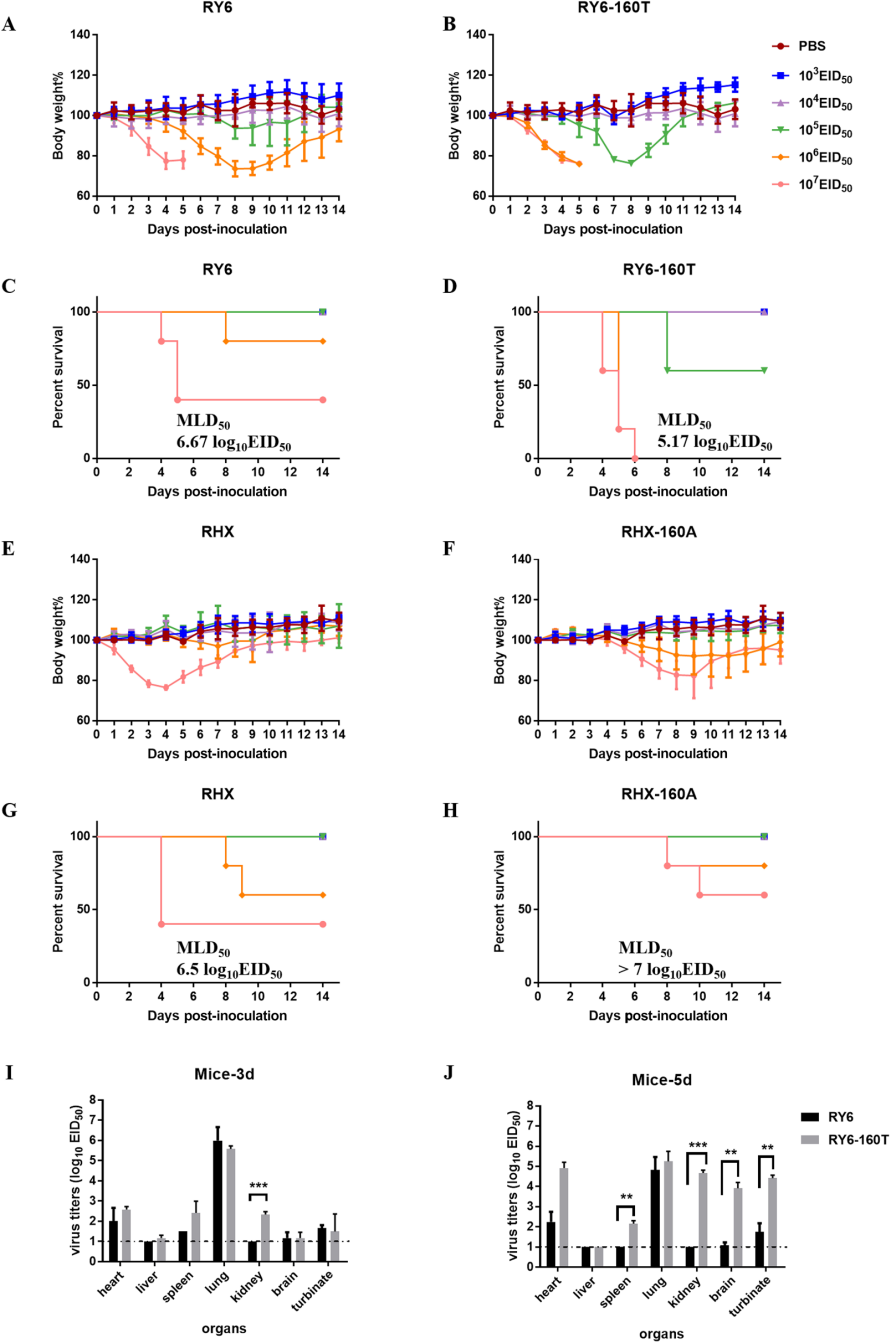
**Pathogenicity of four H5N6 viruses in mice and replication of RY6 and RY6-160 T in mice.** Mean weight of mice infected from 10^3.0^ EID_50_/50 μL to 10^7.0^ EID_50_/50 μL of RY6 (**A**), RY6-160T (**B**), RHX (**E**) and RHX-160A (**F**) (n = 5). Survival rate of mice infected with RY6 (**C**), RY6-160T (**D**), RHX (**G**) and RHX-160A (**H**); mice were humanly killed when they lost ≥ 25% of their initial body weight. (**I** and **J**) Six-week-old SPF BALB/c mice (three/group) were inoculated intranasally with 10^6^ EID_50_ of RY6 and RY6-160T, and organs were collected on days 3 and 5 post-infection for virus titration in eggs. Values represent means ± SD from three independent experiments. (**p < 0.01, ***p < 0.001).

We next investigated if glycosylation at 158 contributed to the pathogenicity of the H5N6 viruses in mice. BALB/c mice (6 weeks-old) were infected intranasally with doses of 10^3^ EID_50_ to 10^7^ EID_50_ of the four recombinant viruses. As shown in Figure 1D, mice infected with RY6-160 T died at the doses of 10^5^–10^7^ EID_50_, suggesting moderate pathogenicity of RY6-160T. Meanwhile, the other three strains induced death in mice at high doses of 10^6^ and 10^7^ EID_50_, representing low pathogenicity. Sixty percent of mice infected with the RHX virus with a dose of 10^6^ EID50 survived (Figure 1G), whereas 80% of mice infected with the RY6 and RHX-160A viruses survived (Figure 1D, H). Hence, the presence of glycosylation site 158 in RHX and RY6-160T, produced stronger pathogenicity and caused disease symptoms and mortality in mice more quickly compared with the equivalent without glycosylation at site 158 (Figure 1B, E). These observations suggest that glycosylation at 158 in RHX and RY6-160 T increased the pathogenicity of viruses.

To further confirm the difference in pathogenicity of the recombinant viruses in mice, we examined virus loads and distribution in the mice infected with RY6 and RY6-160T viruses, a pair with greater differences in pathogenicity than the pair of RHX and RHX-160A. On days 3 and 5 post-infection (dpi), the virus titers of these two strains in the lungs were similar. However, the virus titer of the RY6-160T strain was significantly higher in the kidney than that of the RY6 strain (Figure 1I). On day 5 post infection, virus loads of RY6-160T in the spleen, kidney, turbinate, and brain tissues were significantly higher than in that infected with RY6 (Figure 1J). RY6-160T could replicate more effectively in multiple organs than did RY6. The virus loads of these two strains in various organs except lungs correlated well with their pathogenicity.

**Figure 2:**
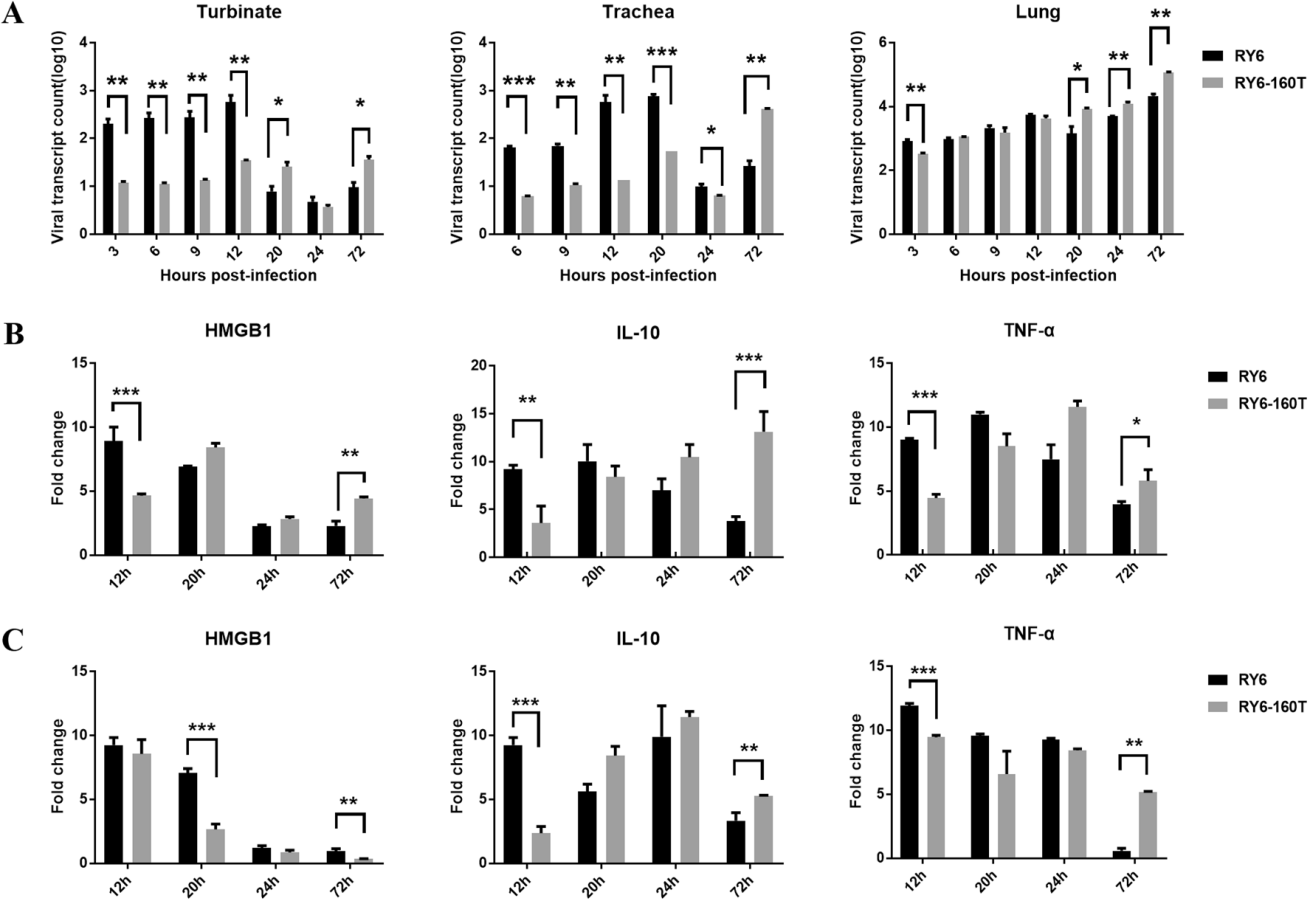
**Viral distribution and cytokine production in respiratory tract of mice.** **A** Six-week-old SPF BALB/c mice (three/group) were inoculated intranasally with 10^6^ EID_50_ of RY6 and RY6-160T, and respiratory tract tissues were collected at 3, 6, 9, 12, 20, 24, 72 hpi for virus titration with RT-qPCR. Relative mRNA levels of HMGB1, IL10, and TNFα in the infected mouse turbinates **B** and lungs **C**. All values were normalized to *Gapdh* and are expressed as fold change compared with controls. Values represent means ± SD from three independent experiments. (*p < 0.05, **p < 0.01, ***p < 0.001).

Since glycosylation at site 158 impacts receptor binding, we quantified virus loads in the airway of mice at an early stage of infection. RY6 replicated faster in the turbinates and trachea than RY6-160 T at 3, 6, 9 and 12 hpi (Figure 2A). However, RY6 virus loads started to decline in nasal turbinates and trachea at 20 hpi. RY6-160T virus loads in all three organs were significantly higher than RY6 on day 3 post-infection (Figure 2A). In both groups, viral loads decreased between 20 and 24 hpi in turbinates and trachea, but increased in lungs with time after infection.

We then conducted RT-PCR to analyze the levels of three cytokines, including high mobility group box 1 protein (HMGB1), interleukin 10 (IL10), and tumor necrosis factor alpha (TNFα) in different tissues. The mRNA levels of all three cytokines were up-regulated in the respiratory tract of RY6 and RY6-160 T virus-infected mice. As shown in Figure 2B, the mRNA levels of the three cytokines in the turbinates of the RY6-infected mice were significantly higher than those infected with the RY6-160T virus. The levels of these three cytokine mRNA peaked at 12–20 hpi. By 72 hpi, HMGB1 mRNA levels were significantly higher in their turbinates of RY6-160T-infected mice than in RY6-infected mice (Figure 2B). This tendency was consistent with the mRNA levels of IL10 and TNFα in the lungs (Figure 2C). HMGB1 expression in the lungs declined gradually over time. At 3 hpi, mice infected with RY6 had higher levels of cytokine expression in the lungs, except for HMGB1.

### Glycosylation at site 158 enhances virus yield in host cells

**Figure 3:**
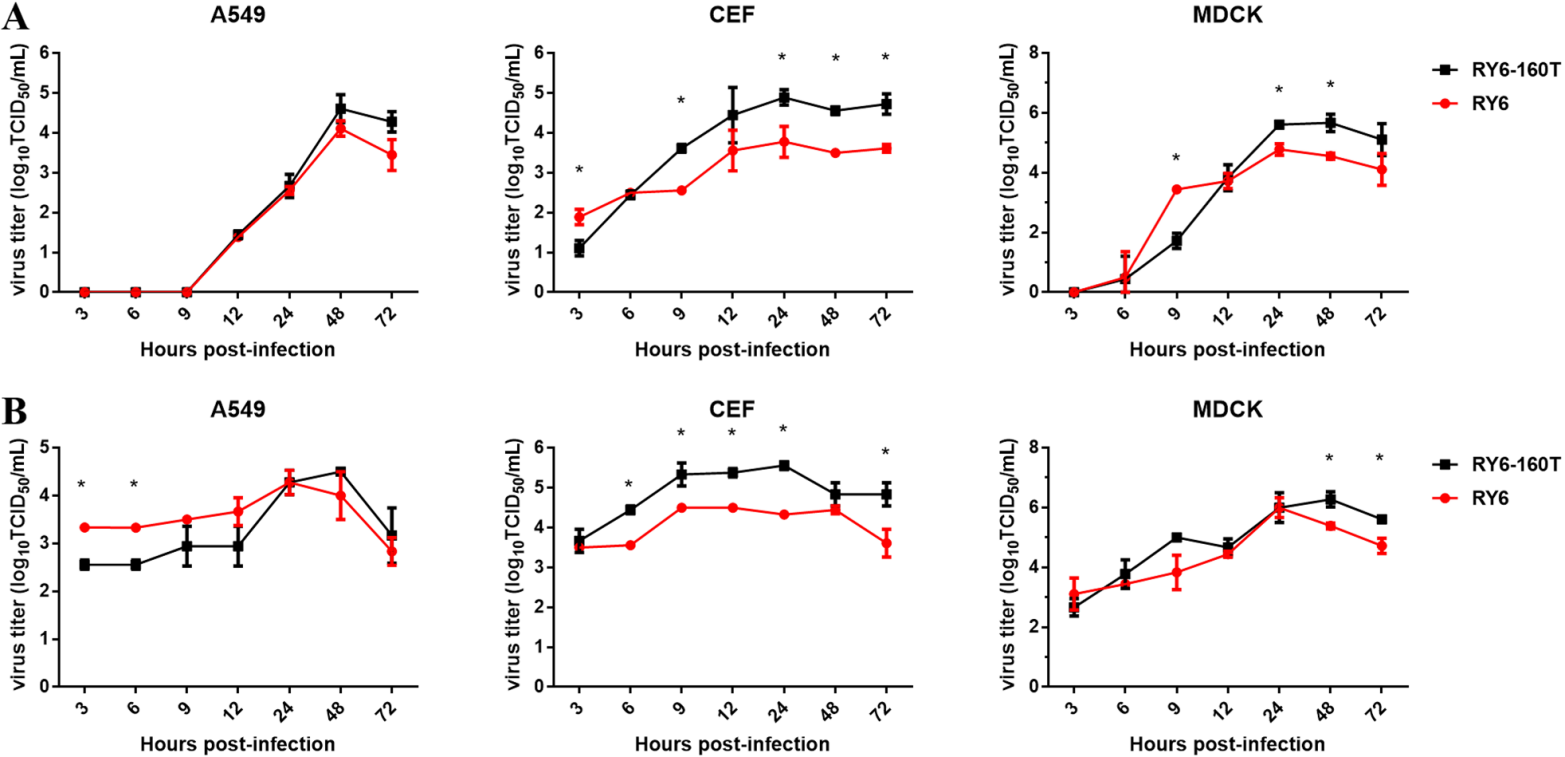
**Growth kinetics of RY6 and RY6-160 T in A549, CEF and MDCK cells.** Growth curves of RY6 and RY6-160 T in different cell lines infected at a multiplicity of infection (MOI) of 0.01 **A** and 5 (**B**). The values represent means ± SD from three independent experiments (*p < 0.05).

We next determined if the difference in the pathogenicity of viruses with or without glycosylation at 158 was due to differential virus replication rates. As shown in Figure 3, A549, CEF, and MDCK cells were infected with RY6 or RY6-160T virus at 0.01 (A) or 5 (B) MOI. The virus titers of RY6 virus in A549 cells infected with 5 MOI were significantly higher than that of RY6-160T virus up to 9 hpi (Figure 3B). In CEF cells, both viruses could be detected at 3 hpi, indicating easier replication in avian cells. Moreover, compared with RY6, RY6-160T showed higher replication efficiency at different time points except at 3 hpi when an MOI of 0.01 was used. MDCK cells were the most suitable for influenza virus replication among the three tested cell lines. The viral titers of the two viruses were the highest in the MDCK cells. RY6 produced higher titers than RY6-160T only at 9 hpi with an MOI of 0.01, but RY6-160T had enhanced replication efficiency late in infection (Figure 3A).

### Glycosylation at site 158 affects receptor binding specificity

**Figure 4:**
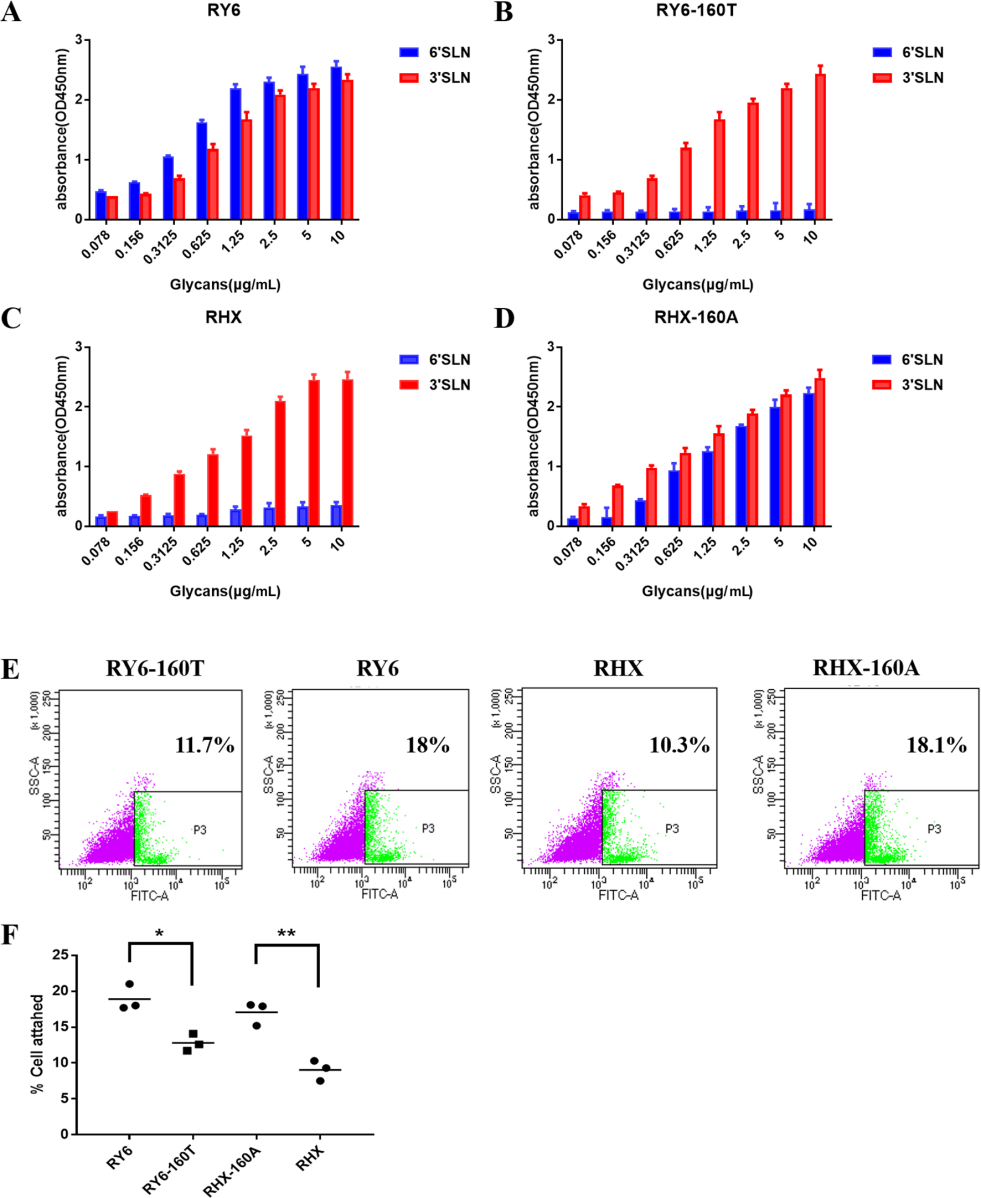
**Solid-phase receptor-binding assay and virus-cell binding assays of four H5N6 viruses.** **A**–**D** The binding of H5N6 viruses to two different biotinylated glycans was tested in a solid-phase binding assay. The data shown are the means of three replicates; the error bars indicate standard deviations. **E** A549 cells were seeded and inoculated at an MOI of 5 with four tested viruses. Virus-bound and unbound cells were analyzed by flow cytometry. **F** Percentages of virus-bound cells as determined by using GraphPad software. Values represent means ± SD from three independent experiments. (*p < 0.05, **p < 0.01).

Given the difference in virus replication both in vitro and in vivo, we evaluated the receptor-binding preferences of the RY6, RY6-160T, RHX, and RHX-160A viruses by using solid-phase binding assays. As shown in Figure 4, all four viruses bound to α2,3SAL (avian-type receptor). RY6 and RHX-160A show higher affinity to α2,6SAL (human-type receptor) (Figures 4A, D). This result indicates that lack of glycosylation at site 158 in HA leads to dual receptor-binding preferences of RY6 and RHX-160A viruses.

### Lack of glycosylation at site 158 contributes to virus binding to A549 cells

Influenza virus infection begins by the binding of the HA protein to the sialic acid receptor of host cells. We determined whether glycosylation site 158 affected virus binding to host cells. A549 cells inoculated with viruses (5 MOI each) of different glycosylation patterns were incubated at 4 °C for 1 h and then analyzed for virus binding by flow cytometry (Figure 4E). The percent of A549 cells bound with RY6 and RHX-160A viruses was much higher than with RY6-160 T and RHX viruses (Figure 4F). These observations suggest that the lack of glycosylation at site 158 facilitated viral attachment to the surface of A549 cells.

### Lack of glycosylation at site 158 enhances the thermostability of the H5N6 virus

**Figure 5:**
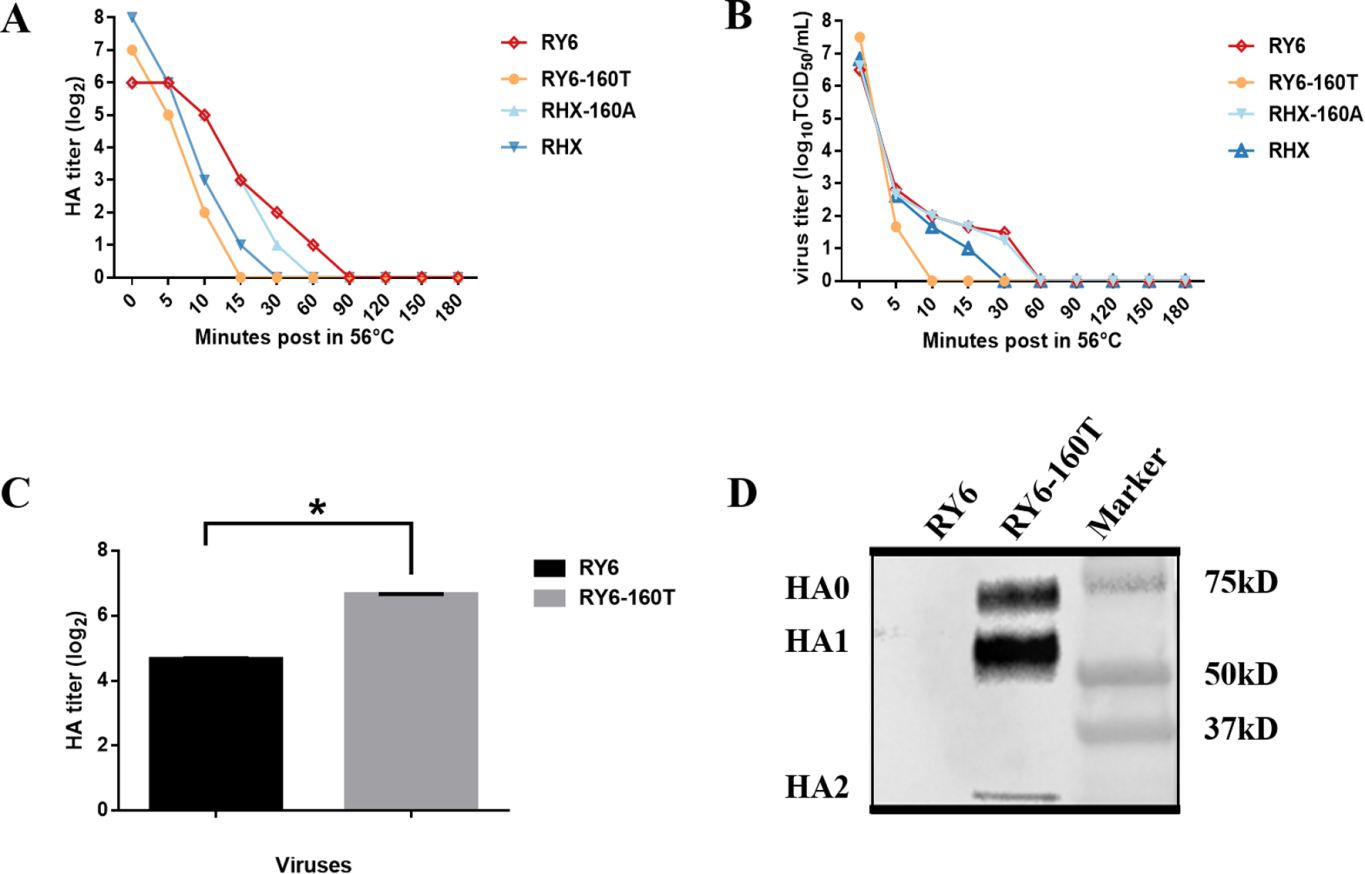
**Thermal stability and virus-like particles (VLP) formation.** The four H5N6 viruses were incubated at 56℃ for 5, 10, 15, 30, 60, 90, 120, 150 and 180 min. **A** Mean HA titers of the four H5N6 viruses were tested by haemagglutination assay. **B** TCID_50_ titers of the four H5N6 viruses were determined in MDCK cells. **C** The supernatants were collected to purify the VLP by sucrose density gradient centrifugation at 48 h, and the hemagglutination test was employed to detect the aboundance of VLP. **D** The HA protein levels were measured in supernatants by western blotting. The values represent means ± SD from three independent experiments (*P < 0.05, ***p < 0.001).

Based on the stability of AIV at 56 °C in water bath, AIV can be categorized as follows: thermal instability, HA titer decreased by 2 titers after 5 min in a 56 °C water bath; Moderate thermal stability, HA titer did not decrease after 5 min but decreased by 2 titers after 30 min; Thermal stability, HA titer decreased by 2 titers after 30 min. We analyzed thermal stability of these four strains and found that all four strains belonged to the category of moderate thermal stability (Figures 5A, B). The HA titer of RY6-160T dropped to 0 within 15 min; whereas the HA titers of RY6 and RHX-160A decreased to 0 after 60 min (Figure 5A). The thermal sensitivity of these four strains was confirmed by their infectivity in cell culture (Figure 5B). Viruses without glycosylation at site 158 were more heat stable.

### The H5N6 virus with glycosylation at site 158 has stable balance between HA binding and NA cleavage

We conducted a hemagglutination assay to evaluate the balance between HA binding and NA cleavage of four H5N6 viruses. At 4 °C, the enzymatic activity of NA was not detectable, whereas HA was able to bind to its receptors and to agglutinate guinea pig red blood cells. At 37 °C, NA became active but the agglutinated erythrocytes became dissociated. The difference in HA titers at 4 °C to 37 °C represent the balance between HA binding and NA cleavage [32]. All four strains had large differences in HA titers after 2 h at 37 °C. HA titers of RY6-160T and RHX viruses dropped to zero but those of RY6 and RHX-160A did not change at either temperature (Table 2). This shows that with the same NA gene fragment, the HA-NA functional balance of RY6 and RHX-160A without glycosylation at site 158 was poor, but that they could bind to guinea pig red blood cells more stably.

**Table 2:** Quantitative assessment of HA affinity and NA cleavability of the four viruses

Viruses	HA titer with 0.8% GPRBC (log2)						Average HA titer loss(log2)
	4 °C, 75 min			37 °C, 2 h			
RY6	5	5	4	5	5	4	0
RY6-160 T	5	5	5	0	0	0	5
RHX	4	4	4	0	0	0	4
RHX-160A	4	4	5	4	4	4	0.33

GPRBC: guinea pig red blood cells.

### Glycosylation at site 158 increases VLP formation

VLP assays are widely used to evaluate the assembly and budding efficiency of influenza virus [33, 34]. We investigated the effect of glycosylation at site 158 of the HA protein on VLP formation by measuring the HA titers and HA protein levels of 293T cells transfected with M1 and NA plus HA derived the RY6 and RY6-160T viruses. As shown in Figure 5C, the HA titers in the conditioned media of 293T cells transfected with RY6-160T cocktail were significantly higher than that from RY6. Also we analyzed the HA protein levels in the cell supernatants (Figure 5D). Compared with RY6, RY6-160T had substantially higher HA protein levels and proteolytic subunits (HA1 and HA2) were observed. These results suggest that glycosylation at site 158 plays an important role in the assembly and budding of the H5N6 virus.

### Lack of glycosylation at site 158 of HA enhances the antiviral responses in A549 cells

**Figure 6:**
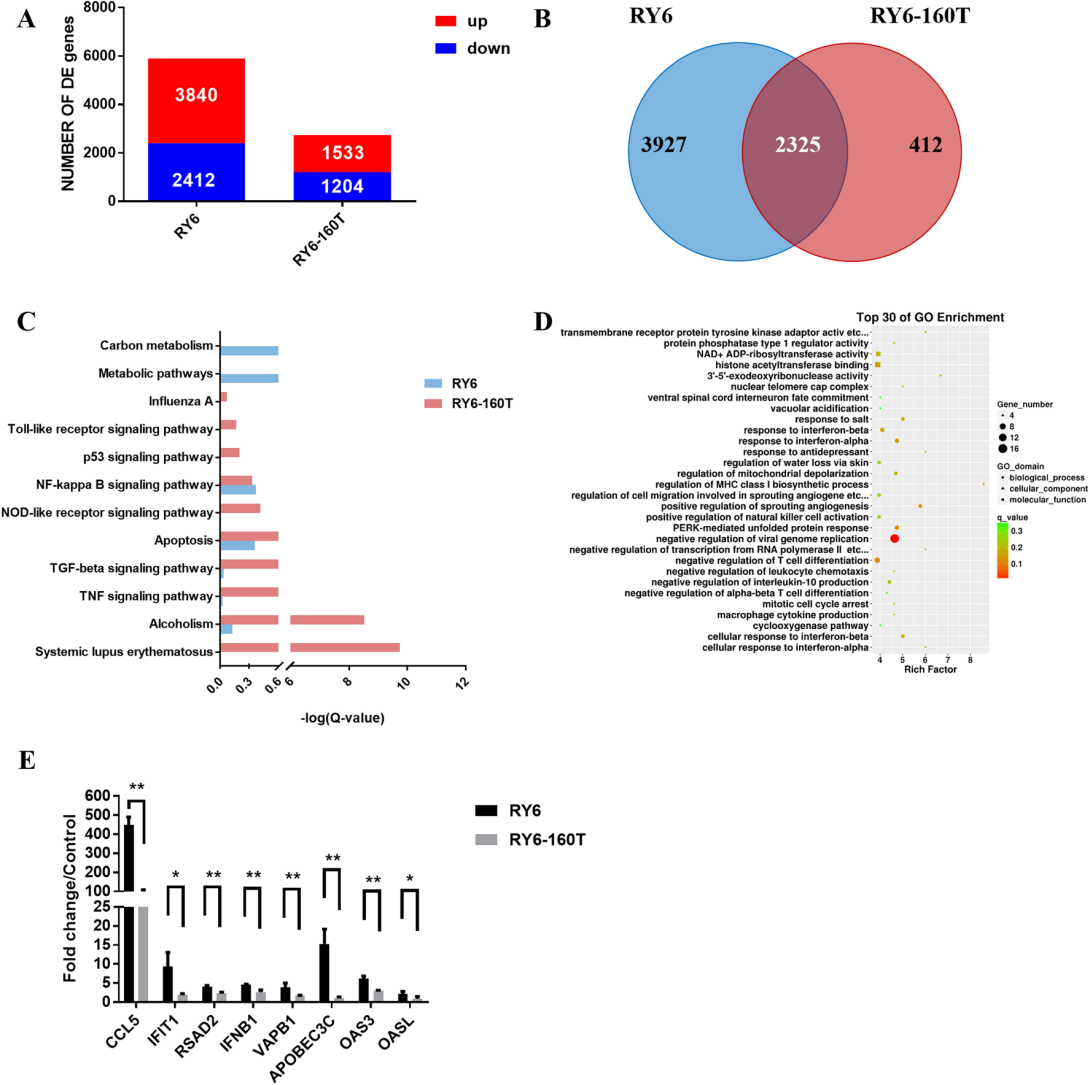
**Transcriptome analysis of differential expression genes (DEG) in RY6- and RY6-160 T-infected A549 cells.** **A** Number of DEG in A549 cells infected with RY6 and RY6-160 T relative to mock-infected A549 cells (q-value <  = 0.05, Fold-change >  = 2). The number of DEG was labeled with color (Red refers to up-regulated, blue refers to down-regulated). **B** Venn diagram showed the distribution of DEG in A549 cells during infection with RY6 and RY6-160 T. **C** The most significantly enriched pathways and classic pathways activated by RY6 and RY6-160 T. **D** Scatter plot of GO enrichment for DEG. The higher the Rich Factor, the higher the degree of enrichment. Q-value represents the *p-value* after multiple hypothesis test correction. The less the value, the higher degree of enrichment. **E** Validation of microarray gene expression data by means of RT-qPCR analysis. All values were normalized to *Gapdh* and are expressed as fold change compared with controls. The values represent means ± SD from three independent experiments. (*p<0.05, **p<0.01, ***p<0.001).

To evaluate the effect of glycosylation at site 158 on the immune response and signal transduction, A549 cells were infected with the RY6 or RY6-160T virus (5 MOI each) and harvested at 6 hpi for transcriptomic analysis. The majority of DEG were up-regulated in A549 cells infected with either virus (Figure 6A). There were 6252 differentially expressed genes in the RY6 group, compared to 2737 genes in the RY6-160T group. Among them, 2325 genes were differentially expressed between the two groups (Figure 6B). These data suggest that the RY6 virus induces DEG more efficiently and diversely. KEGG pathway analysis revealed that the RY6-160T virus preferentially regulated the expression of autoimmune disease-related genes (Figure 6C). In contrast, the RY6 virus preferentially induced DEG mostly enriched in metabolic signaling pathways. Both viruses induced the expression of genes involved in the inflammatory responses. Genes induced in RY6-160T virus-infected cells mostly belong to the cluster of apoptosis as well as the TGFβ and TNF pathways.

GO analysis revealed that differences between the two groups were mainly concentrated in the ‘Biological Process’ category, with the function of the differentially-expressed genes focused on the negative regulation of viral genome replication (Figure 6D). There were 17 genes enriched in this GO term, including Mx, GTPase, ISG15, and the genes in the PKR pathway. These genes inhibit influenza viruses at different stages of the virus life cycle, including endocytosis, replication, and transcription. Eight genes differentially expressed in A549 cells infected with RY6 or RY6-160 T were identified by qRT-PCR, though all of them were upregulated (Figure 6E). Moreover, mRNA levels of antiviral genes in the RY6 group were significantly increased as compared to the RY6-160T group (*p* < 0.05 or *p* < 0.01). These genes except VAPB all belong to the family of interferon stimulating genes (ISG). CCL5, a chemotactic gene that attracts T cells, eosinophils, and basophils into inflammatory sites, was maximally induced in RY6-infected A549 cells, with a fivefold increase (Figure 6E). The landscape of increased gene expression was compatible with the RNA-seq data.

## Discussion

We constructed four recombinant H5N6 viruses based on two wild-type viruses, Y6 and HX, and designated them as RY6 and RY6-160T, and RHX and RHX-160A. The only difference between the pairs is glycosylation site 158 in their HA proteins. We provide evidence that glycosylation site 158 of the HA protein could enhance virulence of H5N6 viruses in mice. Similar to the experiment in vivo, glycosylation site 158 promoted virus replication in vitro. Moreover, the RY6 and RY6-160 T viruses elicited a differential host immune response in A549 cells. These observations collectively suggest that glycosylation at position 158 of the HA protein is a determinant of many biological properties and can trigger a distinct landscape of antiviral innate immunity.

Though it is well received that lack of glycosylation at site 158 enables AIV to preferentially bind the human receptor, whether glycosylation affects the pathogenicity of AIV in mammals remains controversial. Zhang et al. reported that removal of glycosylation at site 158 enhances viral pathogenicity in mice [15]. In contrast, Yen et al. reported that the human reassortant virus VN1203 without glycosylation at site 158 leads to decreased pathogenicity and limited systemic spread in mice [35]. Zhao et al. reported that glycosylation at 158 of H5N1 virus increases virulence in mice but does not alter its receptor binding property [36]. The sialic acid (SA) receptors are differentially expressed in different tissues of the respiratory tract in mice. The SA α2,6-Gal receptor is expressed at high levels in turbinates, while SA α2,3-Gal receptor is predominantly expressed in trachea [37]. In the present study, H5N6 viruses with glycosylation site 158 appeared to be more virulent to mice (Figure 1). But we observed a significant advantage for the replication of RY6 in turbinates, consistent with its dual receptor specificity (Figures 2A and 4A). In addition, the RY6 virus rapidly increased the levels of inflammatory factors over a much shorter period, which potentially made the virulence rather moderate in mice (Figure 2B, C). In contrast, the RY6-160T virus loads peaked at a much later time and increased the levels of inflammatory factors significantly only after 72 hpi. The inflammatory cytokines play an important role in damage to lung tissue [38]. For example, IL10, though it is an anti-inflammatory cytokine, can recruit fibrocytes into the lungs and make scars [39]. These findings indicate that pathogenicity of influenza virus in mice may be related to more than just switch of receptor binding properties.

The functional balance between HA binding and NA cleavage is essential for adaptive evolution of influenza viruses [40, 41]. Glycan at position 158 in the H5N1 HA protein can compensate for reduced enzyme activity of the viral NA and significantly improves elution of the virus from erythrocytes [42]. Our results demonstrate that viruses with glycosylation at site 158 showed functional balance between HA binding and NA cleaving (Table 2). Likewise, RY6-160T displayed a higher efficiency for the assembly of progeny virus (Figure 5C, D). The HA glycoprotein is required for VLP budding and the release of progeny virions from the host cell surface is dependent on the cleavage of sialic acid by NA [43, 44]. We speculate that glycosylation site 158 in the HA protein of RY6-160T may affect virus replication by mediating VLP formation.

According to the global initiative on sharing avian flu data (GISAID), as of 18^th^ March 2020, only 15 of 1294 nucleic acid sequences of H5N6 AIV retain a glycosylation site at position 158 of the HA protein. Therefore H5N6 viruses without a glycosylation site at position 158 show a survival advantage. Heat treatment at neutral pH promotes HA protein fusion, which could serve as a marker of HA stability [45]. Viruses without glycosylation at site 158, which are ubiquitous in nature, could infect host cells after heat treatment over a longer time. In support of this notion, Imai et al. reported that addition of an N158D mutation to an HA protein containing N224K/Q226L increases virus stability [14]. Furthermore, we provide evidence that viruses without glycosylation at site 158 bound to host cells preferentially and replicated faster during early infections in vitro.

The life cycle of influenza viruses involves multiple steps that are tightly regulated by host factors [46]. Host immune response is one of the most important determinants of the pathogenicity of a virus. Our present study shows that RY6 had higher titers during the early stage of infection both in A549 cells and in the mouse respiratory tract but deaccelerated over longer periods of time. RY6-infected A549 cells induced a large number of ISG to robustly antagonize viral infection, in agreement with the growth curve in A549 cells (Figure 6E). Remarkably, the most significantly DEG in the RY6-160T virus-infected cells were associated with autoimmune diseases induced by overexpression of inflammatory factors, which may explain, in part, the increased lethality of RY6-160T in mice. How N-linked glycosylation at site 158 affects host immune responses remains to be investigated.

Taken together, we provide experimental evidence that glycosylation at site 158 of H5N6 HPAIV facilitates VLP formation and enhances virulence in mice in part by inducing a stronger inflammatory process. In contrast, the H5N6 virus lacking N-linked glycosylation at this site was more thermo-stable and induced a stronger antiviral innate immune response that will likely restrict virus replication. Our study suggests that N-glycosylation at site 158 of the HA protein influences a broad spectrum of the biological properties of the H5N6 virus and profoundly impacts the induction of pro-inflammatory and antiviral innate immune responses.

## Data Availability

All data generated or analyzed during this study are included in this published article.

## Abbreviations

A: alanine
AIV: avian influenza virus
A549: Human non-small cell lung carcinoma cell line
CCL5: chemokine (C–C motif) ligand 5
CEF: chicken embryonic fibroblasts
DEGs: differential expression genes
EID_50_: 50% embryo infectious dose
HA: hemagglutinin
HMGB1: high mobility group box 1
HPAIV: highly pathogenic avian influenza virus
IL10: interleukin 10
MLD_50_: mouse median lethal dose
MOI: multiplicity of infection
N: asparagine
NA: neuraminidase
qRT-PCR: quantitative real time PCR
T: threonine
TNFα: tumor necrosis factor alpha
VLP: viral-like particle
